# A prognostic 10‐lncRNA expression signature for predicting the risk of tumour recurrence in breast cancer patients

**DOI:** 10.1111/jcmm.14556

**Published:** 2019-08-20

**Authors:** Jianing Tang, Jiangbo Ren, Qiuxia Cui, Dan Zhang, Deguang Kong, Xing Liao, Mengxin Lu, Yan Gong, Gaosong Wu

**Affiliations:** ^1^ Department of Thyroid and Breast Surgery Zhongnan Hospital of Wuhan University Wuhan China; ^2^ Department of Biological Repositories Zhongnan Hospital of Wuhan University Wuhan China; ^3^ Department of Thyroid and Breast Surgery, Tongji Hospital Huazhong University of Science and Technology Wuhan China; ^4^ Department of General Surgery Zhongnan Hospital of Wuhan University Wuhan China

**Keywords:** breast cancer, GEO, lncRNA, nomogram, prognosis

## Abstract

Breast cancer is one of the most frequently diagnosed malignancies and a leading cause of cancer death among females. Multiple molecular alterations are observed in breast cancer. LncRNA transcripts were proved to play important roles in the biology of tumorigenesis. In this study, we aimed to identify lncRNA expression signature that can predict breast cancer patient survival. We developed a 10‐lncRNA signature‐based risk score which was used to separate patients into high‐risk and low‐risk groups. Patients in the low‐risk group had significantly better survival than those in the high‐risk group. Receiver operating characteristic analysis indicated that this signature exhibited excellent diagnostic efficiency for 1‐, 3‐ and 5‐year disease‐relapse events. Moreover, multivariate Cox regression analysis demonstrated that this 10‐lncRNA signature was an independent risk factor when adjusting for several clinical signatures such as age, tumour size and lymph node status. The prognostic value of risk scores was validated in the validation set. In addition, a nomogram was established and the calibration plots analysis indicated the good performance and clinical utility of the nomogram. In conclusion, our results demonstrated that this 10‐lncRNA signature effectively grouped patients at low and high risk of disease recurrence.

## 1 INTRODUCTION

Breast cancer is one of the most frequently diagnosed malignancies and a leading cause of cancer death among females around the world, accounting for 24% of diagnosed cancer and 15% of cancer death in females. According to *Global Cancer Statistics 2018*, there will be nearly 2.1 million new cases diagnosed globally, with approximately 62 000 deaths. The incident rates of breast cancer increased in most developing countries during last decades, resulting from a combination of social and economic factors, including the postponement of childbearing, obesity and physical inactivity.^1^ In the developed countries, the incidence of breast cancer is markedly higher. Nearly 60% of deaths occur in the developing counties. It is a major health burden in both developed and developing countries. Prognosis of patients with breast cancer has been improved as a result of recent advances of radiotherapy, hormone therapy, chemotherapy and immunotherapy. However, quite a few patients diagnosed and treated at early stages will unfortunately suffer from locoregional or distant tumour recurrence months or years later.^2^, ^3^

Breast cancer is a heterogeneous disease, and it is widely acknowledged that inheritance plays important roles in the initiation and progression of breast cancer. Multiple molecular alterations are observed in breast cancer. It was reported that 5%‐10% of breast cancer cases resulted from hereditary and genetic factors, such as inherited mutations and family history.^1^ BRCA mutations occur in 20% triple‐negative breast cancer patients, whereas in the general population, the mutations of BRCA are less common. To date, BRCA1 and BRCA2 mutations are currently detected to assess the risk of inherited breast cancer.^4^

In order to predict recurrence and mortality of breast cancer, previous studies stratified patients into high‐ and low‐risk groups based on their histopathological features, including tumour size, lymph node status and grade.^5^ While because of molecular differences, clinical outcomes are largely different even in patients with histologically similar tumours.^6^ During the past decade, molecular studies demonstrated that there were at least four molecular subtypes of breast cancer: luminal, basal, human epidermal growth factor receptor 2 (HER2)‐enriched and normal‐like. These subtypes exhibit different histopathological features and treatment sensitivities.^7^ Patients with luminal breast cancer often have a better prognosis, whereas those with HER2‐enriched or basal‐like types have a poorer prognosis. For HER2‐positive breast cancers, the monoclonal antibody, trastuzumab and the dual tyrosine dual kinase inhibitor, lapatinib, were approved.^8^, ^9^, ^10^, ^11^ Because of the heterogeneity of breast cancer, multiple gene prognostic signatures could provide further prognostic information, and several molecular prognostic profiles have been validated for clinical use.^12^ The 21‐genes score (Oncotype DX) calculates a recurrence score and divides breast tumours into low‐, intermediate‐ and high‐risk groups to estimate the likelihood of distant recurrence in tamoxifen‐treated patients with oestrogen receptor‐positive breast cancer.^13^, ^14^, ^15^ The Amsterdam 70‐gene signature accurately grouped patients into low or high risk to predict distant metastases and deaths.^16^, ^17^ Detection of these biomarkers alone or in combination assists early diagnosis, therapeutic strategies determination and prognosis prediction after treatment.

Analysis of mammalian transcriptomes demonstrated that more than 50% of transcripts have no protein‐coding potential. Long non‐coding RNA (lncRNA) is a subset of these non‐coding transcripts >200 nucleotides.^18^ Accumulating evidence indicated that lncRNAs were involved in cancer progression. In breast cancer, several lncRNAs were associated with the prognosis and indicated their potential roles in prediction of clinical outcome.

In the present study, we constructed a multi‐lncRNA‐based signature and developed a nomogram to predict the relapse‐free survival (RFS) survival of patients with breast cancer. Our findings suggested that this multi‐lncRNA‐based signature could be used as an effective prognostic predictor for patients with breast cancer.

## 2 MATERIALS AND METHODS

### 2.1 Data processing and differentially expressed lncRNAs screening

The GSE21653 data set was downloaded from the GEO database (https://www.ncbi.nlm.nih.gov/geo/) which contains 266 breast cancer cases. This data set was based on GPL570 platform ([HG‐U133_Plus_2] Affymetrix Human Genome U133 Plus 2.0 Array). Patients without complete information of size, lymph node status, grade, oestrogen receptor status, progesterone receptor status, HER2 and survival status were excluded from this study. A total of 227 patients (71 with recurrence disease and 156 without recurrence) were selected for further analysis. Probes were annotated by the annotation files. Robust Multi‐array Average (RMA) algorithm in affy package in R was used to pre‐process the gene expression profile data. After background correction, quantile normalization and probe summarization, the expression value of each gene was compared between recurrence samples and recurrence‐free ones to identify differentially expressed lncRNAs (DELs) by Linear Models for Microarray Data (LIMMA) package. *P*‐value <.05 and |log2 fold‐change (FC)| > 2 were set as the cut‐off criteria to select genes for further analysis.

### 2.2 Construction of the lncRNA‐based prognostic signature

After screening out the DELs, we carried out univariate Cox regression analysis to identify prognostic lncRNAs. A *P* value <.05 was considered as significant. Lasso‐penalized Cox regression was then performed to narrow the lncRNAs for prediction of the RFS.^19^ The LASSO Cox regression model was analysed using the ‘glmnet’ package. LASSO shrinks all regression coefficients towards zero and sets the coefficients of many irrelevant features exactly to zero base on the regulation weight *λ*. The optimal *λ* was chosen according to minimum cross‐validation error in 10‐fold cross‐validation. Finally, a multivariate Cox regression analysis was conducted to assess the contribution of a lncRNA as an independent prognostic factor for patient survival. A stepwise method was employed to select the best model, and a risk score was calculated with the coefficients weighted by the penalized Cox model in the training set. The optimal cut‐off of risk score was obtained using ‘survminer’ package in R. All patients were classified into either high‐risk or low‐risk group based on the optimal cut‐off of risk score.

### 2.3 Construction of the nomogram

A nomogram was constructed using the ‘rms’ R package. Calibration plots were performed to assess the prognostic accuracy of the nomogram. The predicted outcomes and observed outcomes of the nomogram were presented in the calibrate curve, and the 45° line represents the best prediction.

### 2.4 External data validation

To further validate the predictive value of the signature, we analysed the data set GSE19615 and GSE20685 with a total of 115 and 327 cases, respectively. These two data sets were based on platform GPL570 ([HG‐U133_Plus_2] Affymetrix Human Genome U133 Plus 2.0 Array).

### 2.5 Statistical analysis

To investigate the prognostic accuracy of multi‐lncRNA‐based classifier, time‐dependent receiver operating characteristic (ROC) analysis was performed using the ‘survivalROC’ R package. Relapse‐free survival was analysed based on Kaplan‐Meier method, and the log‐rank test was performed to assess the statistical significance of the differences between different groups. Cox regression model was used to analyse multivariable survival analysis. Hazard ratios (HR) with their respective 95% confidence intervals were obtained. A *P* value <.05 was considered statistically significant, and all tests were two‐sided. All statistical tests were performed with R software (Version 3.5.0).

### 2.6 Gene set enrichment analysis

A total of 227 breast cancer samples in GSE21653 were divided into two groups (high risk vs low risk) according to the optimal cut‐off of risk scores. In order to identify the significantly alerted Kyoto Encyclopedia of Genes and Genomes (KEGG) pathways, we performed gene set enrichment analysis (GSEA) between the high‐risk and low‐risk groups using the Java GSEA implementation. Annotated gene set c2.cp.kegg.v6.2.symbols.gmt (Version 6.2 of the Molecular Signatures Database) was chosen as the reference gene set. FDR <0.05 was chosen as the cut‐off criteria.

## 3 RESULTS

### 3.1 Analysis of DELs

**Figure 1:**
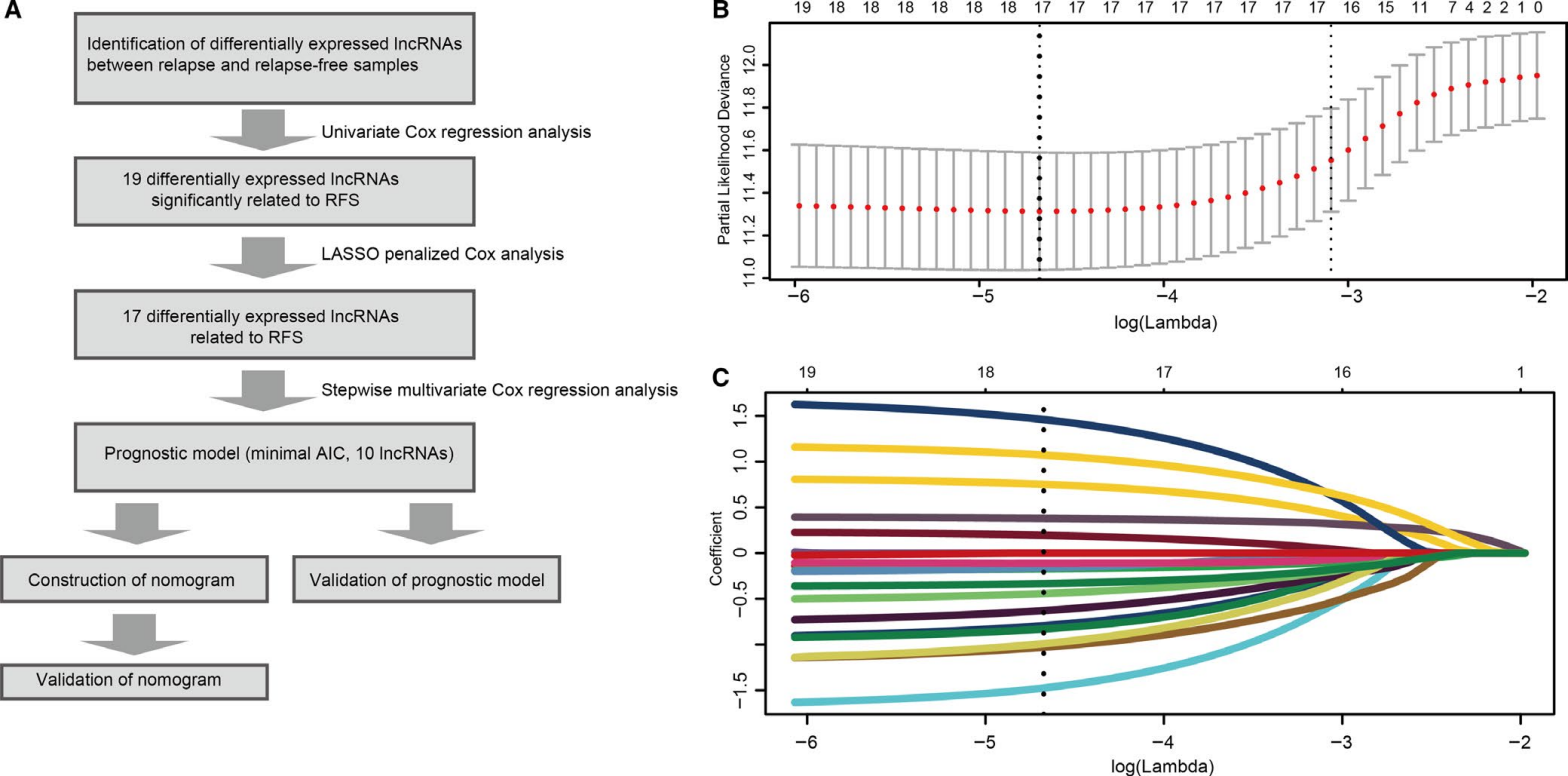
Flow chart and 10‐time cross‐validation for tuning parameter selection. A, Flow chart indicating the process used to select target genes included in the analysis. B, Ten‐time cross‐validation for tuning parameter selection in the lasso model. C, LASSO coefficient profiles of the 19 prognostic lncRNAs. A vertical line is drawn at the value chosen by 10‐fold cross‐validation

A flow chart of the analysis procedure was developed to describe our study (Figure 1). In the presented study, 71 disease‐relapse samples and 156 disease‐relapse free samples in the data set of GSE21653 were analysed. Based on the cut‐off criteria of *P*‐value <.05 and |log2 fold‐change (FC)| > 2, a total of 30 DELs were identified, including nine up‐regulated and 21 down‐regulated DELs. Univariate Cox regression analysis was performed to identify prognostic lncRNAs. The patients were stratified into high expression and low expression groups according to optimal cut‐off of each lncRNA. The 19 lncRNAs significantly associated with the RFS were considered as prognostic lncRNAs for further analysis.

### 3.2 Patient characteristics

The clinicopathologic characteristics of patients in the training set were shown in Table S1. The median follow‐up in training set was 5.04 years (low‐risk group) and 3.02 years (high‐risk group). In the validation set GSE19615, median follow‐up was 5.9 years (low‐risk group) and 4.3 years (high‐risk group). In the validation set GSE20685, median follow‐up was 8.1 years (low‐risk group) and 6.75 years (high‐risk group). Fifty‐seven (63.3%, training set), 12 (33.3%, validation set GSE19615) and 52 (37.3%, validation set GSE20685) patients in the high‐risk group developed relapse during the follow‐up period.

### 3.3 Identification of a multi‐lncRNA‐based signature

**Figure 2:**
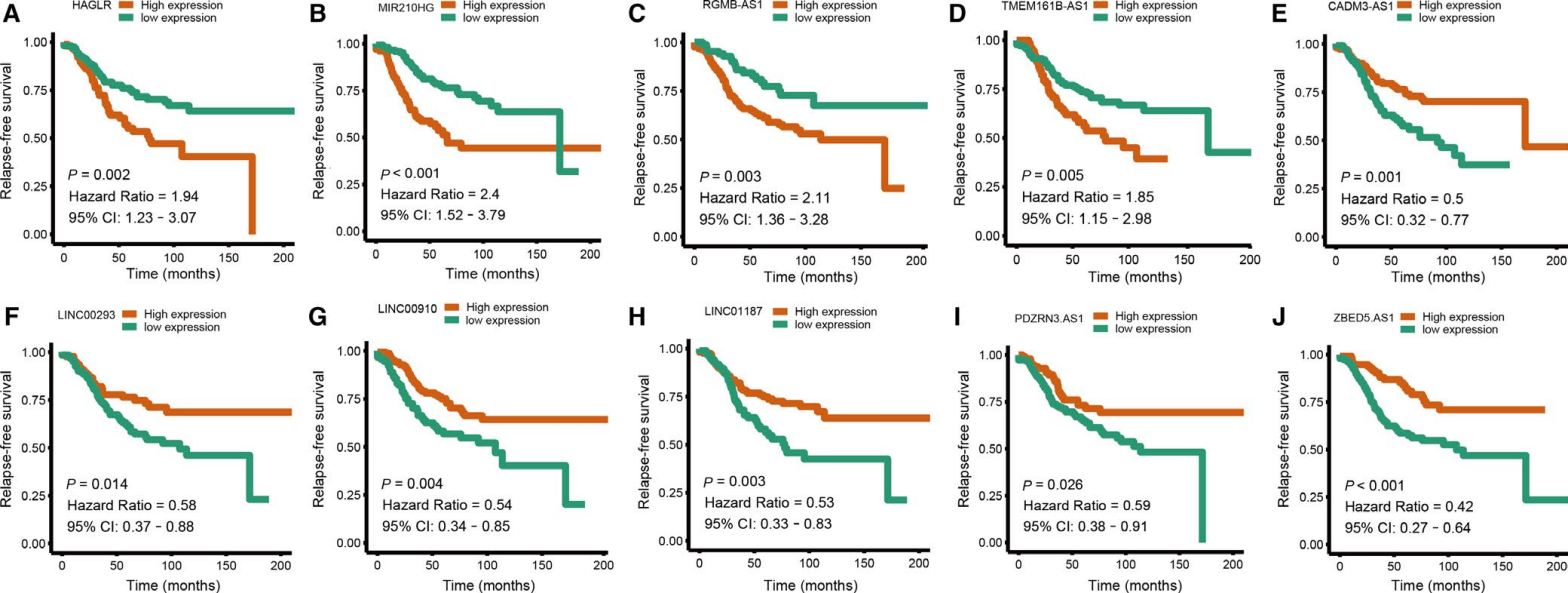
Univariate Cox regression analysis of the ten prognostic lncRNAs in the signature. A, HAGLR. B, MIR210HG. C, RGMB‐AS1. D, TMEM161B‐AS1. E, CADM3‐AS1. F, LINC00293. G, LINC00910. H, LINC01187. I, PDZRN3‐AS1. J, ZBED5‐AS1

**Figure 3:**
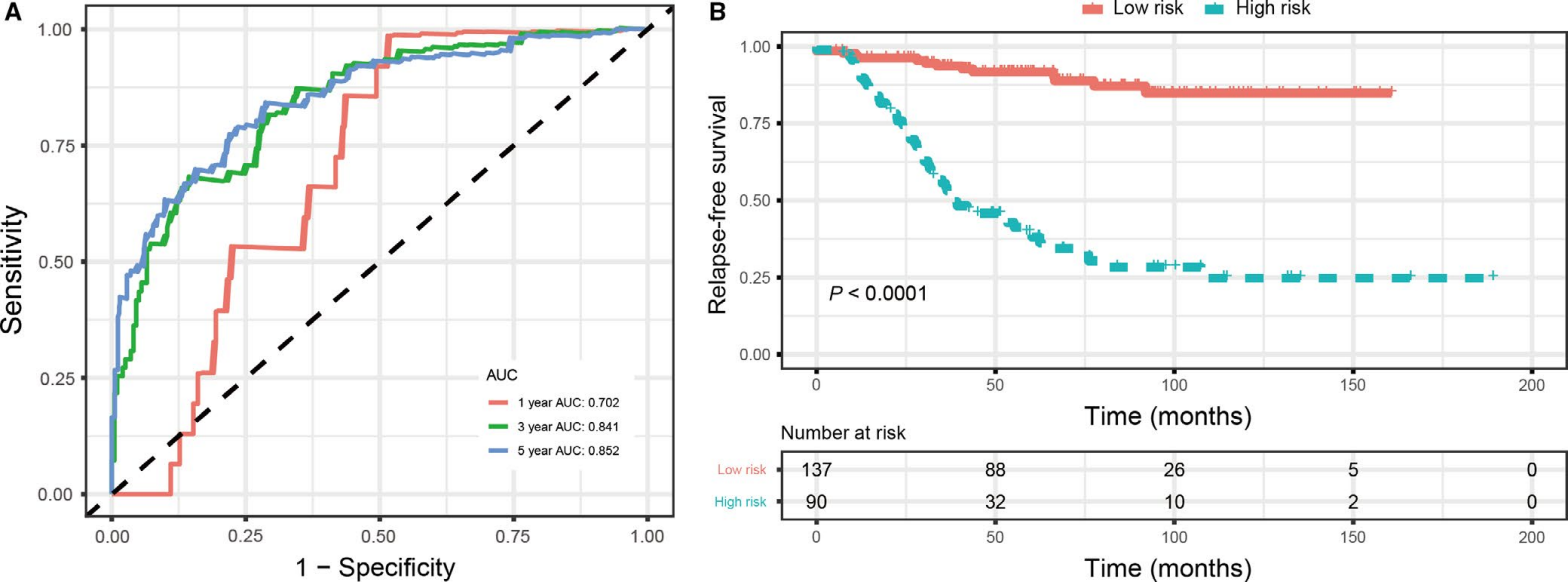
Validation of prognostic risk score model in training set. A, Time‐dependent receiver operating characteristic curves of the 10‐lncRNA signature. B, Kaplan‐Meier survival analysis of the 10‐lncRNA signature

**Figure 4:**
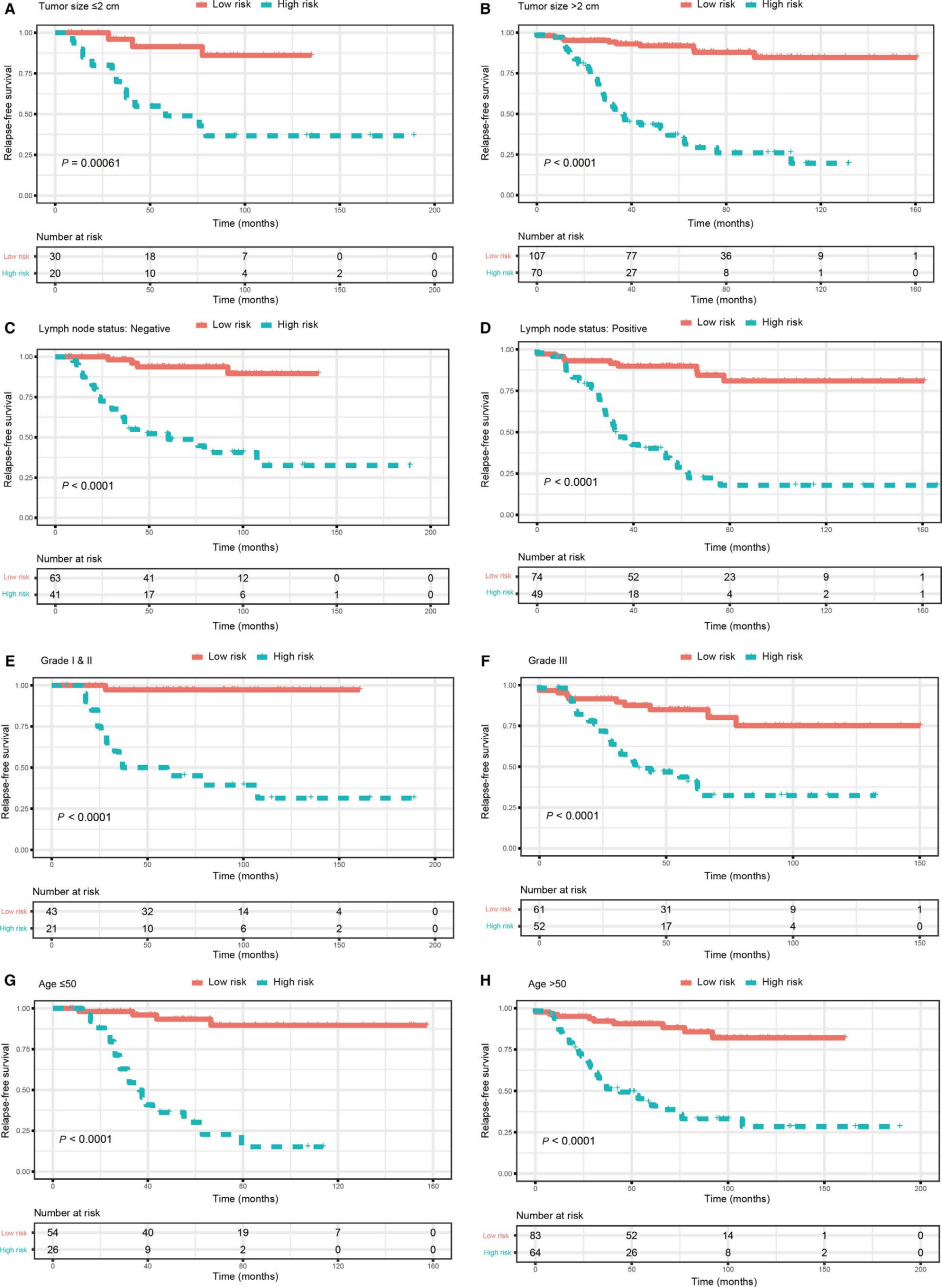
Kaplan‐Meier survival analysis for patients according to the 10‐lncRNA‐based signature stratified by clinicopathological risk factors. A, B, Tumour size. C, D, Lymph node status. E, F, Tumour grade. G, H, Age

After primary filtration of univariate Cox regression which identified 19 lncRNAs significantly associated with the RFS, a Lasso‐penalized Cox analysis with 10‐fold cross‐validation was performed to narrow the lncRNAs for prediction of the RFS. As a result, 17 lncRNAs were identified. Subsequently, a stepwise multivariate Cox regression analysis was conducted, and 10 lncRNAs were finally identified as prognostic lncRNAs to build a predictive model. This predictive model was defined as the linear combination of the expression levels of the 10 lncRNAs weighted by their relative coefficient in the multivariate Cox regression model, as risk score = (−1.02 × expression of CADM3‐AS1) + (0.91 × expression of HAGLR) + (−1.19 × expression of LINC00293) + (−1.79 × expression of LINC00910) + (−2.09 × expression of LINC01187) + (0.62 × expression of MIR210HG) + (−0.57 × expression of PDZRN3‐AS1) + (1.83 × expression of RGMB‐AS1) + (1.30 × expression of TMEM161B‐AS1) + (−0.28 × expression of ZBED5‐AS1). Among these 10 prognostic lncRNAs, HAGLR, MIR210HG, RGMB‐AS1 and TMEM161B‐AS1 showed positive coefficients in the Cox regression analysis, indicating their high expression levels for shorter RFS. CADM3‐AS1, LINC00293, LINC00910, LINC01187, PDZRN3‐AS1 and ZBED5‐AS1 showed negative coefficients, suggesting that their high expression levels were associated with better RFS. These results were consistent with the previous univariate Cox regression analysis (Figure 2). The optimum cut‐off score was generated by using ‘survminer’ package in R via the maximally selected rank statistics. Patients with a risk score of −6.63 or higher were in the high‐risk group, and the others were in the low‐risk group. Patients in the lower‐risk group had significantly better DFS than those in the high‐risk group (Figure 3). The prognostic accuracy of the 10‐lncRNA‐based signature was assessed by calculating the AUCs of a time‐dependent ROC curve at 1, 3 and 5 years. Higher AUC indicated better prognostic performance. In the training set, AUCs of the 10‐lncRNA‐based signature were 0.702, 0.841 and 0.852 at 1‐, 3‐ and 5‐year survival times, respectively, indicating that the prognostic model had a high sensitivity and specificity (Figure 3). Multivariate Cox proportional hazards regression analysis demonstrated that the 10‐lncRNA signature was an independent risk factor when adjusting for the classical clinicopathologic factors (Table S2). When the patients were stratified by clinicopathological risk factors, the 10‐lncRNA signature was still a statistically significant prognostic model for patients in the high‐risk group with poorer prognosis (Figure 4).

### 3.4 Validation of the signature

**Figure 5:**
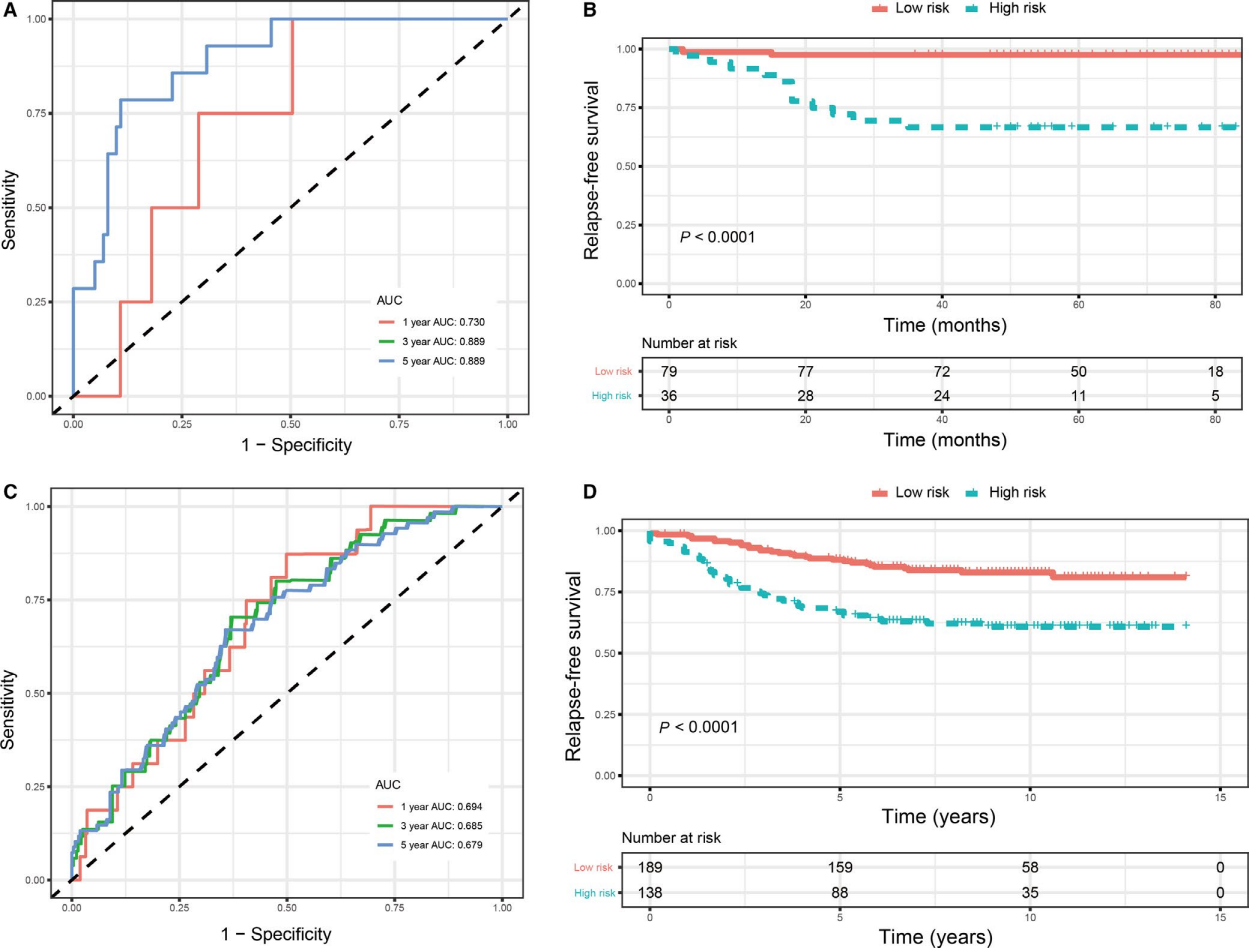
Validation of 10‐lncRNA signature in validation sets. A, Time‐dependent receiver operating characteristic (ROC) curves of the 10‐lncRNA signature in GSE19615. B, Kaplan‐Meier survival analysis of the 10‐lncRNA signature in GSE19615. C, Time‐dependent ROC curves of the 10‐lncRNA signature in GSE20685. D, Kaplan‐Meier survival analysis of the 10‐lncRNA signature in GSE20685

To further assess the predictive value of this 10‐lncRNA signature, two external validation sets (GSE19615 and GSE20685) were used to validate our results. According to the 10‐lncRNA‐based signature identified above, patients with breast cancer in these two validation sets were divided into a high‐ and a low‐risk groups (based on the threshold of −6.63). Compared with the high‐risk ones, significantly higher survival rates were observed in the low‐risk group (Figure 5), which was consistent with the results from the training set. ROC curve indicated good prognostic performance in both GSE19615 and GSE20685. In GSE19615, AUCs at 3 years were the same as that at 5 years, and no patients relapsed during the 2 years. Multivariate Cox proportional hazards regression analysis also demonstrated that the 10‐lncRNA signature was an independent risk factor (Table S2).

### 3.5 Nomogram development

**Figure 6:**
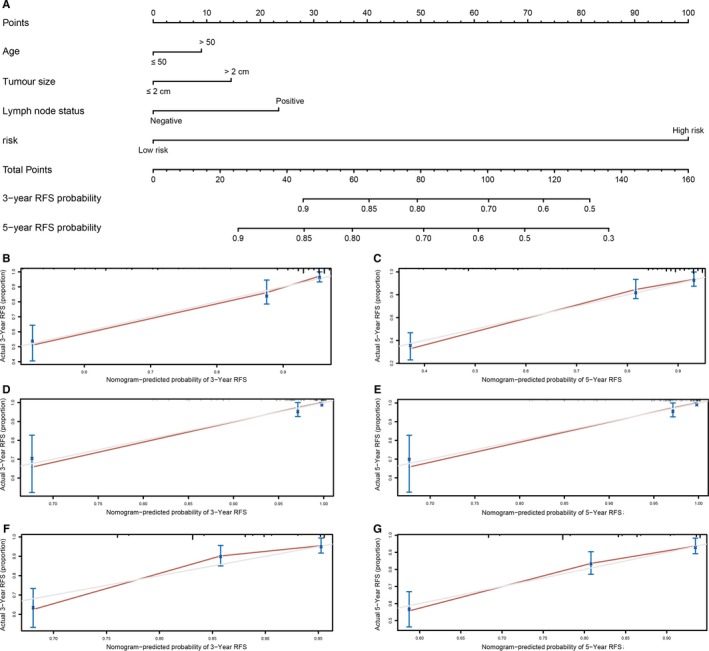
Nomogram to predict risk of cancer recurrence. A, Nomograms to predict risk of cancer recurrence. B, 3‐y nomogram calibration curves of training set. C, 5‐y nomogram calibration curves of training set. D, 3‐y nomogram calibration curves of validation set GSE19615. E, 5‐y nomogram calibration curves of validation set GSE19615. F, 3‐y nomogram calibration curves of validation set GSE20685. G 5‐y nomogram calibration curves of validation set GSE20685

To predict the recurrence probability of patients with breast cancer using a quantitative method, we constructed a nomogram that integrated both the 10‐lncRNA‐based signature and the conventional clinicopathological factors (Figure 6A) to predict 3‐ and 5‐year DFS probability. Calibration plots indicated that the nomogram had good accuracy as an ideal model both in training set and validation set (Figure 6B‐G).

### 3.6 Gene set enrichment analysis

**Figure 7:**
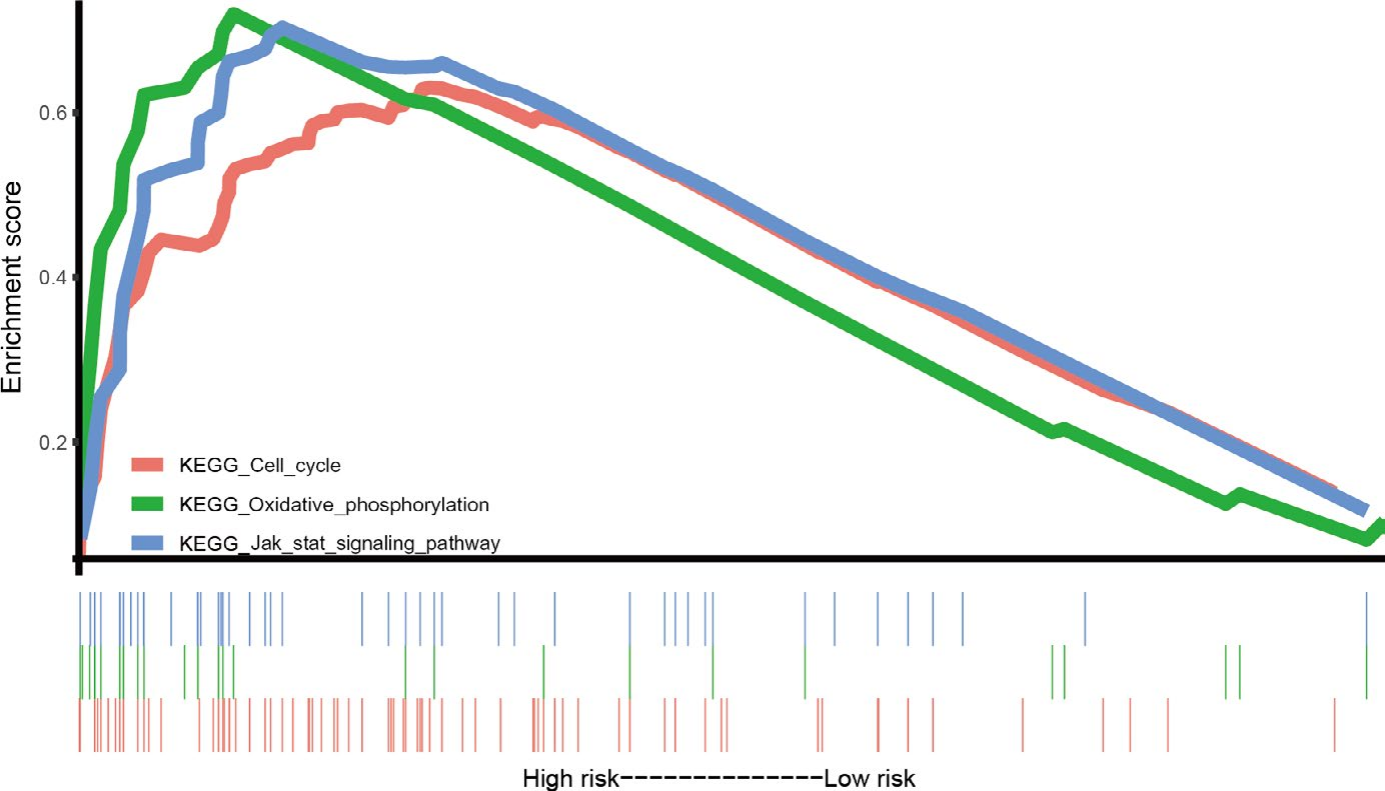
Gene set enrichment analysis

To identify the significant changes of biological pathways between high‐ and low‐risk groups, the GSEA was performed. Based on the cut‐off criteria of FDR <0.05, three significantly altered pathways were selected: cell cycle pathway, oxidative phosphorylation pathway and JAK/STAT signalling pathway (Figure 7).

## 4 DISCUSSION

Breast cancer accounts for most frequent malignant tumours and cancer death in females around the world. Quite a few patients still suffer from locoregional or distant tumour recurrence even after combined therapies. Inheritance modulates the development of breast cancer, and various genetic changes were found to regulate breast cancer initiation and progression. In breast cancer, lncRNA transcripts were proved to play important roles in the biology of tumorigenesis, whereas the prognostic significance of lncRNAs was not well investigated. So far, many biomarkers have been identified for the diagnosis and treatment of breast cancer. However, most of these studies only focused on one or a few genes, and few systemic investigations were carried out for clinical application of these genes.

In the presented study, we constructed and validated a 10‐lncRNA‐based signature (HAGLR, MIR210HG, RGMB‐AS1, TMEM161B‐AS1, CADM3‐AS1, LINC00293, LINC00910, LINC01187, PDZRN3‐AS1 and ZBED5‐AS1) to predict RFS for patients with breast cancer. The GSE21653 data set was used to identify DELs between disease‐relapse samples and disease‐relapse free ones. After univariate, Lasso and multivariate Cox analysis, we eventually selected 10 lnRNAs to construct a multi‐gene signature for prognosis prediction. This 10‐lcnRNA signature was assessed in the training set. Patients in the low‐risk group had significantly better survival than those in the high‐risk group. ROC analysis showed that this signature exhibited excellent diagnostic efficiency for the 1‐, 3‐ and 5‐year disease‐relapse event. Moreover, using multivariate Cox regression model, the 10‐lncRNA signature was proved to be an independent risk factor when adjusting for several clinical signatures such as age, tumour size and lymph node status. When patients were stratified by clinicopathological features, the 10‐lncRNA‐based signature remains a strong prognostic model. Similar results were also observed in the external validation set. These results demonstrated that this 10‐lncRNA signature could successfully categorize patients into high‐risk and low‐risk groups with different RFS and was an effective prognostic indicator for patients with breast cancer.

To date, several nomograms and prognostic models have been constructed to predict the prognosis of patients with breast cancer. Rouzier developed and validated a nomogram, which was based on oestrogen receptor status, clinical stage, histologic grade and number of pre‐operative chemotherapy cycles to predict distant metastasis‐free survival.^20^ Cheng et al developed a robust 4‐gene signature (SRPK1, PCCA, PRLR and FBP1) to predict distant relapse‐free survival (DRFS) for patients with HER2‐negative breast cancer following taxane and anthracycline‐based chemotherapy. It was proved to be more accurate than other clinical signatures, such as tumour size, lymph node invasion and TNM stages.^21^ Liu et al^22^ constructed a lncRNA signature to predict ER‐positive breast cancer metastasis following tamoxifen treatment, but the sample size was limited in this study. A 42‐gene classifier was also constructed to predict ER‐positive breast cancer recurrence.^23^ The Oncotype DX (21 genes), the Amsterdam 70‐gene signature (70 genes) and the Risk of Recurrence Score (ROR, 50 genes) derived from PAM50 are the three most commonly used molecular prognostic profiles. However, their clinical applicability was restricted because of high cost. Previous studies have constructed prognostic models using lasso and multivariate Cox regression analysis. Long et al established a four‐gene‐based prognostic model to predict overall survival in patients with hepatocellular carcinoma. The four‐gene‐based prognostic model was constructed based on 356 hepatocellular carcinoma patients obtained from TCGA and was validated using only one external data set (78 patients).^19^ And DNA methylation sites were also used for construction of models to predict survival of patients. Dong et al constructed a model using three risk categories (low risk, intermediate risk and high risk) to predict the overall survival of patients with hepatocellular carcinoma based on 134 methylation sites. Cox regression, SVM‐RFE and FW‐SVM algorithms were used to screen out differentially methylated sites. And this study was performed based on TCGA (training set) and GSE77269 (validation set), the sample size of our study was limited, and large‐scale cohort studies are needed.^24^ In this study, we constructed a signature involving only 10 lncRNAs. Multivariate Cox regression demonstrated that the prognostic value of the 10‐lncRNA signature was independent of age, tumour size and lymph node status. A nomogram was then developed to integrate both the 10‐lncRNA‐based signature and clinicopathological risk factors to accurately predict the likelihood of RFS in patients with breast cancer. Calibration plots indicated that the actual RFS corresponded closely with predicted RFS, suggesting our nomogram had good predictive performance both in the training and validation sets.

HAGLR, also known as HOXD‐as1, was involved in the occurrence and progression of variate types of human tumours, including bladder cancer, hepatocellular carcinoma, prostate cancer, gastric cancer, neuroblastoma and lung cancer.^25^, ^26^, ^27^, ^28^, ^29^, ^30^, ^31^ In prostate cancer, HOXD‐AS1 recruited WDR5 to mediate histone H3 lysine 4 tri‐methylation, thus promoting cell proliferation, chemo‐resistance and castration resistance.^28^ In ovarian cancer, HOXD‐AS1 was reported to competitively bind to miR‐608 to regulate the expression of frizzled family receptor 4 (FZD4) and to enhance proliferation, migration and invasion capabilities of ovarian cancer cells.^32^ MIR210HG was significantly up‐regulated in glioma tissues than tumour‐adjacent normal tissues. The serum levels of MIR210HG levels were also significantly higher in glioma patients compared with healthy controls.^33^ Based on public database analysis, MIR210HG served as a biomarker or a therapeutic target in colorectal adenocarcinoma.^34^ RGMB‐AS1 was reported to play important roles in lung cancer progression, the expression levels of which were significantly correlated with differentiation, TNM stage and lymph node metastasis. RGMB‐AS1 promoted cell proliferation, migration and invasion capabilities of lung cancer and thyroid papillary cancer.^35^, ^36^ RGMB‐AS1 was down‐regulated as an independent favourable prognostic factor for hepatocellular carcinoma patients.^37^ The biological function of the remaining lncRNAs (TMEM161B‐AS1, CADM3‐AS1, LINC00293, LINC00910, LINC01187, PDZRN3‐AS1 and ZBED5‐AS1) in our signature has not been investigated in previous studies; thus, further studies are required to investigate the underlying molecular mechanisms of these diagnostic lncRNAs.

The high‐throughput platforms for genomic analysis provided promising tools in medical oncology with great clinical applications. Although it is difficult to use such a large number of genes for clinical application, accumulating studies indicated that lncRNAs were involved in cancer progression. The prognostic significance of lncRNAs has not been well investigated. In the presented study, we developed a prognostic signature based on 10 lncRNAs expression and constructed a novel nomogram to predict the RFS. These findings might lead to the development of a cheap molecular test and suitable in the clinical routine. Although the nomogram demonstrated an accurate survival prediction, several limitations should not be ignored. The sample size of our study was limited, and large‐scale cohort studies are performing to investigate the prognostic value of this 10‐lncRNA signature. As only the patients who had complete information were included in our study, there might be a selection bias in the primary cohort. Several predictors, such as radiotherapy and Ki‐67 index, were not analysed. In addition, the biological functions of the 10 lncRNAs in breast cancer progression are to be revealed. Our study only included the data set based on GPL570 platform, not representing all possible lncRNAs. The underlying mechanisms of these lncRNAs in our signature remain largely unclear. Further in vivo and in vitro studies are required to confirm the exact molecular mechanisms of these diagnostic genes.

In conclusion, our results demonstrated that the 10‐lncRNA signature effectively grouped patients at low and high risk of disease relapse. Thereby, it may be a useful predictive tool with a good prospect of clinical application for patients with node‐positive breast cancer.

## CONFLICT OF INTEREST

The authors declare that the research was conducted in the absence of any commercial or financial relationships that could be construed as a potential conflict of interest.

## AUTHOR CONTRIBUTIONS

JT, ML, YG and GW reviewed relevant literature and drafted the manuscript. DZ, DK, XL, JR and QC conducted all statistical analyses. All authors read and approved the final manuscript.

## ETHICAL APPROVAL

The research was carried out according to the World Medical Association Declaration of Helsinki and was approved by the Ethics Committee at Zhongnan Hospital of Wuhan University. Patients used in this manuscript were extracted from the GEO registry database. Informed consent was not applicable.

## Data Availability

Data sharing is not applicable to this article as no new data were created or analyzed in this study.
