# Supplementary material for: A prognostic 10‐lncRNA expression signature for predicting the risk of tumour recurrence in breast cancer patients
Source: J Cell Mol Med. 2019 Aug 20;23(10):6775–84. doi: 10.1111/jcmm.14556 (PMC6787455; doi:10.1111/jcmm.14556)
Supplement: Supplementary file 1 [file JCMM-23-6775-s001.docx]

| **Table S1. Clinicopathologic characteristics of three sets of breast cancer patients according to the integrated lncRNA signature** | | | | | | |
| --- | --- | --- | --- | --- | --- | --- |
| **Variables** | **Training set GSE21653 (n=227)** | | **Validation set GSE19615 (n=115)** | | **Validation set GSE20685 (n=327)** | |
|  | **Low risk (%)** | **High risk (%)** | **Low risk (%)** | **High risk (%)** | **Low risk (%)** | **High risk (%)** |
| **Age at diagnosis (years)** |  |  |  |  |  |  |
| **Median** | 54 | 53 | 53 | 60 | 47 | 46 |
| **≤50** | 54(39.4) | 26(28.9) | 17(47.2) | 26(28.9) | 123(65.1) | 86(62.3) |
| **>50** | 83(60.6) | 64(71.1) | 19(52.8) | 64(71.1) | 66(34.9) | 52(37.7) |
| **Tumor size** |  |  |  |  |  |  |
| **≤2 cm** | 30(21.9) | 20(22.2) | 14(38.9) | 20(22.2) | 75(39.7) | 26(18.8) |
| **>2cm** | 107(78.7) | 70(77.8) | 22(61.1) | 70(77.8) | 114(60.3) | 112(81.2) |
| **Lymph node status** |  |  |  |  |  |  |
| **Negative** | 63(46.0) | 41(45.6) | 17(47.2) | 41(45.6) | 91(48.1) | 46(33.3) |
| **Positive** | 74(54.0) | 49(54.4) | 19(52.8) | 49(54.4) | 98(51.9) | 92(66.7) |
| **Grade** |  |  |  |  |  |  |
| **I** | 28(20.4) | 11(12.2) | 3(8.3) | 11(12.2) |  |  |
| **II** | 48(35.0) | 27(30.0) | 10(27.8) | 27(30.0) |  |  |
| **III** | 61(44.5) | 52(57.8) | 23(63.9) | 52(57.8) |  |  |
| **ER status** |  |  |  |  |  |  |
| **Negative** | 56(40.9) | 44(48.9) | 15(41.7) | 44(48.9) |  |  |
| **Positive** | 81(59.1) | 46(51.1) | 21(58.3) | 46(51.1) |  |  |
| **PR status** |  |  |  |  |  |  |
| **Negative** | 67(48.9) | 47(52.2) | 17(47.2) | 47(52.2) |  |  |
| **Positive** | 70(51.1) | 43(47.8) | 19(52.8) | 43(47.8) |  |  |
| **HER2 status** |  |  |  |  |  |  |
| **Negative** | 126(92.0) | 78(86.7) | 20(55.6) | 78(86.7) |  |  |
| **Positive** | 11(8.0) | 12(13.3) | 16(44.4) | 12(13.3) |  |  |
| **Disease-relapse event** | 14 | 57 | 2 | 12 | 31 | 52 |
| **Median Follow-up (years)** | 5.04 | 5.9 | 4.3 | 3.02 | 8.1 | 6.75 |

| **Table S2. Multivariate Cox proportional hazards regression analysis of the clinicopathologic characteristics and integrated RNA signature with RFS** | | | | | | | |  |
| --- | --- | --- | --- | --- | --- | --- | --- | --- |
| **Variable** | **Training set GSE21653** | | **Validation set GSE19615** | | **Validation set GSE20685** | | |  |
|  | **HR (95%Cl)** | **P** | **HR (95%Cl)** | **P** | | **HR (95%Cl)** | **P** |  |
| **Age (≦50 vs. >50 y)** | 1.029(0.615,1.721) | 0.913 | 1.758(0.569,5.426) | 0.327 | | 1.225(0.817,1.838) | 0.327 |  |
| **Tumor size (≦2 vs. >2 cm)** | 1.249(0.691,2.260) | 0.462 | 1.448(0.425,4.928) | 0.554 | | 1.056(0.606,1.840) | 1.840 |  |
| **Lymph node status (Negative vs. Positive)** | 1.674(1.016,2.756) | 0.043 | 1.839(0.579,5.842) | 0.302 | | 3.743(2.100,6.673) | <0.001 |  |
| **Integrated RNA signature (low risk vs. high risk)** | 9.008(4.969,16.331) | <0.001 | 12.892(3.082,62.620) | 0.001 | | 2.565(1.636,4.024) | <0.001 |  |
